# Repeat hepatectomy versus thermal ablation therapy for recurrent hepatocellular carcinoma: a systematic review and meta-analysis

**DOI:** 10.3389/fonc.2024.1370390

**Published:** 2024-03-28

**Authors:** Renhua Dong, Ting Zhang, Wenwu Wan, Hao Zhang

**Affiliations:** ^1^ Department of Hepatobiliary and Pancreatic Surgery, Meishan People’s Hospital, Meishan, Sichuan, China; ^2^ Department of Gastroenterology, Meishan People’s Hospital, Meishan, Sichuan, China

**Keywords:** repeat hepatectomy, thermal ablation therapy, meta-analysis, recurrent hepatocellular carcinoma, systematic review

## Abstract

**Background:**

This meta-analysis was conducted to assess the survival benefits of repeat hepatectomy (RH) and thermal ablation therapy (TAT) in managing recurrent hepatocellular carcinoma (HCC).

**Methods:**

A comprehensive search was conducted in the PubMed, SinoMed, Embase, Cochrane Library, Medline, and Web of Science databases using relevant keywords to identify all studies published on this specific topic. Pooled odds ratios (ORs) with corresponding 95% confidence intervals (CIs) were estimated using a fixed-effects model.

**Results:**

This meta-analysis included a total of 21 studies, comprising 2580 patients with recurrent HCC, among whom 1189 underwent RH and 1394 underwent TAT. Meta-analysis results demonstrated that the RH group exhibited superior overall survival (OS) (HR=0.85, 95%CI 0.76∼0.95, P=0.004) and recurrence-free survival (RFS) (HR=0.79, 95%CI 0.7∼0.9, P<0.01) compared to the TAT group. Regarding postoperative complications, the TAT group experienced fewer complications than the RH group (OR=3.23, 95%CI 1.48∼7.07, P=0.003), while no significant difference in perioperative mortality was observed between the two groups (OR=2.11, 95%CI 0.54∼8.19, P=0.28).

**Conclusion:**

The present study demonstrates that, in comparison to TAT, RH may confer superior survival benefits for patients with recurrent HCC.

## Introduction

The postoperative recurrence rate of hepatocellular carcinoma (HCC) is significantly high, with an incidence exceeding 50% at 3 years and surpassing 70% at 5 years (1). Therefore, it is crucial to develop an effective strategy for managing recurrent HCC in order to improve patient survival. Salvage liver transplantation is considered the primary therapeutic approach for patients with recurrent HCC due to its comprehensive consideration of excising cancerous tissue and addressing the entire cirrhotic liver, thereby offering patients the most promising prospects for survival (2). However, it should be noted that this treatment option’s feasibility is severely limited by donor scarcity, restricting its applicability and benefits to a select group of patients (3, 4). Consequently, repeat hepatectomy (RH) and thermal ablation therapy (TAT) have emerged as viable alternative treatment modalities for individuals experiencing recurrent HCC (5, 6).

Although hepatectomy is regarded as the gold standard for the treatment of HCC (7), RH is controversial in the treatment of recurrent HCC due to the excessive damage to liver function caused by surgical resection and the extremely difficult to reoperation (8). As a minimally invasive and repeatable treatment, TAT is currently considered a good choice for treating recurrent HCC (9). Several published studies have compared the effectiveness of these two surgical methods in the treatment of recurrent liver cancer, but there is still controversy in terms of survival. Numerous studies have conducted comparative analyses of the efficacy between repeat hepatectomy and thermal ablation therapy in the treatment of recurrent liver cancer (10–12). However, controversy remains surrounds the question of which surgical approach is more effective in significantly prolonging patient survival. Several meta-analyses on this topic have been published, but these studies exhibit certain methodological concerns (13, 14). For instance, in the meta-analysis conducted by Liu et al (15) and Yang et al (14), the comparison of prognoses between the two patient groups was based on 1-year or three-year survival rates, overlooking the situations of patients lost to follow-up or censored. Additionally, Yuan et al. (13) meta-analysis included data that were not appropriately matched or corrected for multiple factors. These factors may contribute to bias in the results of the meta-analysis. Simultaneously, new research on the treatment of recurrent liver cancer using these two surgical modalities has been published (16–18). Given these circumstances, there is a necessity to update the meta-analysis on this topic. Therefore, this study conducts a meta-analysis to explore the clinical efficacy of repeat hepatectomy and thermal ablation therapy in treating recurrent liver cancer.

## Methods

### Search strategies

This study adhered to the Preferred Reporting Items for Systematic Reviews and Meta-Analyses (PRISMA) guidelines (19). Two investigators (H.Z. and T.Z.) independently conducted a comprehensive literature search on the treatment of recurrent HCC using TAT and RH. The search was performed in PubMed, Embase, Cochrane Library, Medline, and Web of Science databases utilizing relevant MESH terms and free-text variations such as (“repeat hepatectomy” OR “repeat liver resection” OR “repeat hepatic resection”) and (“thermal ablation” OR “radiofrequency ablation” OR “microwave ablation” OR “ablation”) and (“recurrent hepatocellular carcinoma” OR “recurrent HCC” OR “HCC recurrence”). No restrictions were imposed on publication date or journal category. The literature search included articles published in English and Chinese before December 31, 2023. Additionally, we thoroughly examined the reference lists of identified studies to identify any relevant publications that might have been overlooked. While meta-analyses are commonly employed to evaluate controversies in randomized controlled trials (RCTs), they can also be applied to retrospective studies. To ensure more robust conclusions, our analysis included both randomized controlled trials and comparable retrospective studies.

### Inclusion and exclusion criteria

The inclusion criteria were as follows: (1) patients diagnosed with recurrent HCC who underwent RH (open or laparoscopy), with a comparison group undergoing TAT. (2) ensured comparability in baseline patient characteristics across the included studies. (3) outcome measures should encompass survival data, including but not limited to overall survival (OS), recurrence-free survival (RFS), and other relevant metrics. Conversely, the exclusion criteria include: (1) studies lacking a control group for comparison. (2) materials presented solely in the form of case reports, abstracts, conference presentations, or those involving animal experiments. (3) incomplete full-text articles where the abstract fails to provide comprehensive information about the study.

### Data extraction and quality assessment

The article selection and data extraction were conducted by two authors (R.H. and T.Z.). In case of any disagreement regarding the inclusion or exclusion of an article, consultation with the author (H.Z.) was sought for resolution. Following completion of data extraction, a thorough review was performed by the author (H.Z.), and in case of any discrepancies, the data were re-extracted for subsequent analysis and discussion. The extracted information from included studies encompassed details such as first author, publication date, study design, number of cases, age distribution, gender composition, overall survival rate, recurrence-free survival rate, major morbidity rates and mortality rates. In addition, all included studies were evaluated for quality using the ROBINS-I tool.

### Data synthesis and analysis

The meta-analysis was conducted using RevMan 5.3 software, a Cochrane-endorsed tool for systematic reviews. Dichotomous variables were assessed utilizing the odds ratio (OR) and a 95% confidence interval (CI) as statistical measures for effect analysis. Hazard Ratio (HR) was used to analyze overall survival (OS) and recurrence-free survival (RFS). In cases where explicit HR values were not provided in the literature, we applied the method of Parmar et al. (20) to extract HR values. Heterogeneity within included studies was examined using the Mantel-Haenszel test with I^2^ values categorized as follows: low heterogeneity when I^2^ ≤ 25%, moderate heterogeneity when 25% < I^2^ ≤ 50%, and high heterogeneity when I2 > 50%. A fixed-effects model was used under conditions of low or moderate heterogeneity; otherwise, a random-effects model was adopted. Sensitivity analysis employing the one-out method was conducted to assess our findings’ robustness, while a funnel plot based on primary outcomes served as an evaluation tool for publication bias in this study. Throughout all analyses, statistical significance is considered at P value <0.05 for overall effect.

## Results

### Search results

**Figure 1:**
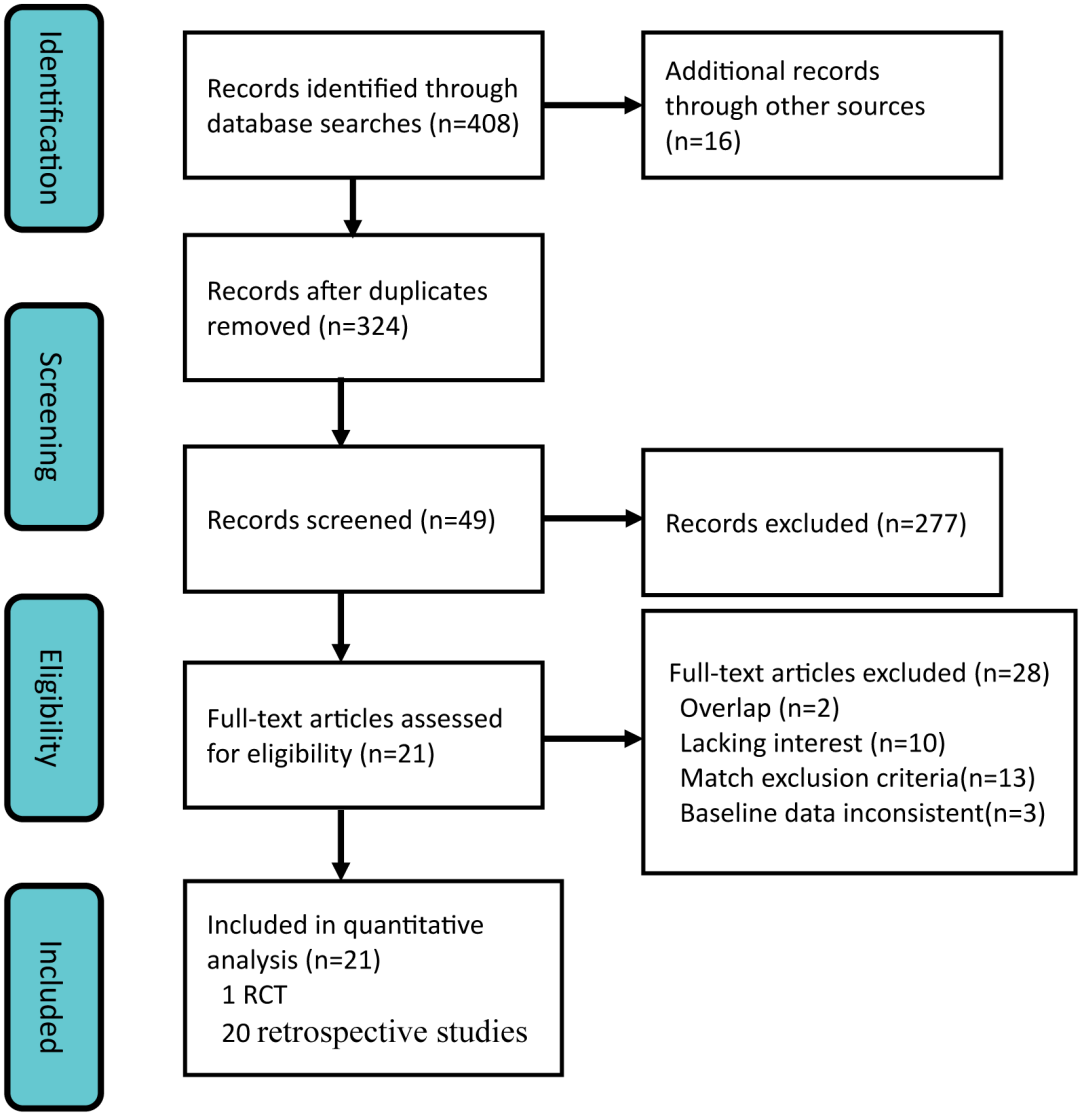
PRISMA flow chart of the literature selection.

The flow diagram illustrating the search results is presented in **Figure 1**. Following the devised retrieval strategy, a total of 324 relevant references were identified after eliminating duplicates. After reviewing the titles and abstracts, 49 articles with potential relevance were retained. Among these, 28 studies were excluded during full-text analysis due to reasons such as overlapping centers or patient cohorts (2 studies), lack of significant outcomes (10 studies), meeting one or more exclusion criteria (13 studies) and baseline data inconsistent (21–23). Ultimately, a meta-analysis was conducted on a selected set of 21 studies (10–12, 16–18, 24–38), comprising one randomized controlled trial (12) and twenty retrospective studies.

### Characteristics and quality of included studies

The basic characteristics of the included studies are presented in **Table 1**. All publications spanned from 2007 to 2023 and encompassed a total cohort of 2580 patients, including 1186 patients in the RH group and 1394 patients in the TAT group. Of these 21 studies, 16 were conducted in China (including Hong Kong and Taiwan), 3 in Japan and 2 in Korea. In the included studies, there was no statistically significant difference in baseline data (such as tumor size and number of tumors, etc.) between the two groups. Both RH and TAT groups in each study were from the same single or multiple centers during the same period. The ROBINS-I tool was used to assess the quality of the 21 included studies, and the specific results are shown in **Supplementary Table S1**.

**Table 1:** Characteristics of the included studies.

Author	Year	Country	Study design	Patients (n)	Age (mean ± SD)		Sex (M/F)		Child–Pugh (A/B)		Tumor size (mean ± SD,cm)		Tumor number (single/multiple)	
					RH	TAT	RH	TAT	RH	TAT	RH	TAT	RH	TAT
Choi	2007	Korea	N-RCT	23	NA	NA	NA	NA	NA	NA	NA	NA	NA	NA
Liang	2008	China	N-RCT	110	48.8 ± 12.0	54.6 ± 10.8	39/5	54/12	44/0	64/2	≤3(26)	≤3(44)	34/10	48/18
Ueno	2009	Japan	N-RCT	19	68.0 ± 6.8	68.0 ± 4.3	4/5	10/0	8/1	7/3	1.8 ± 0.38	1.8 ± 0.35	NA	NA
Umeda	2010	Japan	N-RCT	87	64.8 ± 0.79		63/24		29/0	51/7	3.2 ± 0.57	3.1 ± 0.30	18/11	34/24
Chan	2012	China	N-RCT	74	52.0 ± 10.3	59.0 ± 11.0	NA	NA	29/0	40/5	2.1 ± 1.2	2.2 ± 1.3	21/7	29/16
Hirokawa	2011	Japan	N-RCT	31	69.0 ± 7.3	67.0 ± 7.3	8/2	17/4	10/1	21/3	1.9 ± 0.7	1.7 ± 0.6	7/3	16/5
Cheng	2012	China	N-RCT	104	56.3 ± 12.3	61.0 ± 11.1	40/14	39/11	51/3	50/0	2.9 ± 1.8	2.3 ± 1.9	NA	NA
Zhang	2014	China	N-RCT	66	47 ± 13	52 ± 13	25/2	37/2	27/0	37/2	3.2 ± 1.0	2.7 ± 1.1	25/2	32/7
Wang	2015	China	N-RCT	290	50.2 ± 10.1	52.7 ± 10.9	113/15	148/14	NA	NA	2.4 ± 0.9	2.3± 0.7	89/39	107/55
Song	2015	Korea	N-RCT	117^*^	52.5 ± 9.8	53.6 ± 10.9	31/8	58/20	39/0	78/0	NA	NA	32/7	65/13
Chen	2018	China	N-RCT	105	73.5 ± 3.5	73.7 ± 2.9	41/7	51/6	NA	NA	2.6 ± 1.14	2.5 ± 1.2	28/20	30/27
Peng	2018	China	N-RCT	102^*^	55.3 ± 14.3	56.0 ± 14.3	46/5	45/6	48/3	49/2	2.4 ± 1.0	2.4 ± 0.9	43/8	43/8
Xia	2019	China	RCT	240	53.0 ± 8.8		216/24		NA	NA	NA	NA	NA	NA
Xiao	2019	China	N-RCT	35	NA	NA	10/1	18/6	11/0	24/0	NA	NA	5/6	11/13
Feng	2020	China	N-RCT	96^*^	56.6 ± 9.	58.2 ± 7.5	42/6	42/6	47/1	46/2	2.5 ± 0.5	2.5 ± 0.5	37/11	34/14
Lu	2020	China	N-RCT	240^*^	50.3 ± 10.5	50.9 ± 11.6	108/12	104/16	120/0	120/0	2.4 ± 1.1	2.2 ± 1.0	106/14	106/14
Wang	2020	China	N-RCT	71^*^	NA	NA	23/2	40/6	NA	NA	≤3(20)	≤3(39)	19/6	38/8
Zhong	2021	China	N-RCT	454^*^	NA	NA	194/33	191/36	222/5	224/3	<3(128)	<3(135)	171/56	172/55
Shi	2022	China	N-RCT	44^*^	53.2 ± 11.3	55.2 ± 10.0	17/5	17/5	NA	NA	2.9 ± 1.4	3.4 ± 1.3	14/8	15/7
Wang	2023	China	N-RCT	120^*^	52.0 ± 8.9	53.0 ± 14.0	54/6	54/6	NA	NA	2.5 ± 0.4	2.4 ± 0.4	NA	NA
Wan	2023	China	N-RCT	152^*^	56.1 ± 8.7	57.6 ± 8.4	66/10	69/7	63/13	61/15	4.6 ± 2.1	4.9 ± 2.1	62/14	57/19

RH, repeat hepatectomy; TAT, thermal ablation therapy; NA, not available; RCT, randomized controlled trial; NOS, Newcastle-Ottawa scale; SD, standard deviation. *Data after propensity matching scores.

### Overall survival and recurrence free survival

**Figure 2:**
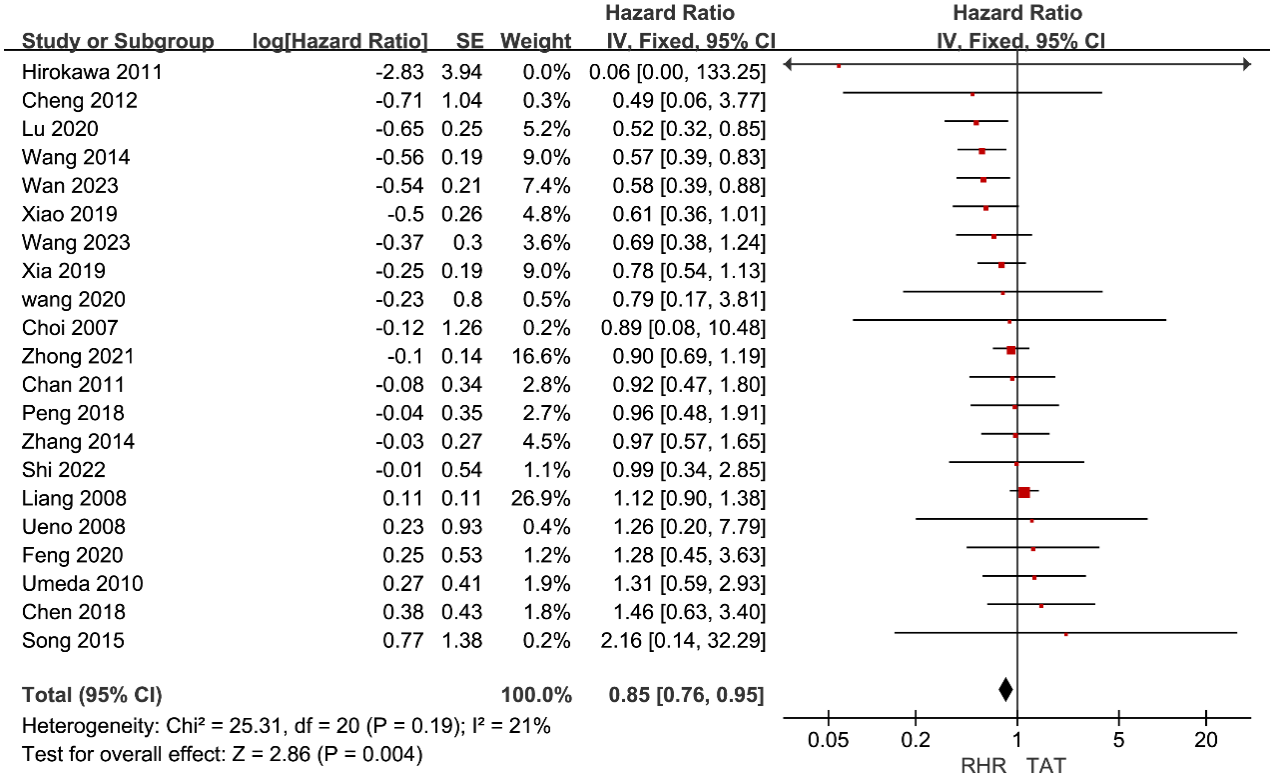
Forest plots of pooled data on overall survival.

The HR values of OS in all included studies (10–12, 16–18, 24–38) were extracted as the effect size for meta-analysis. The heterogeneity among studies was low (I^2 ^= 21%). Therefore, a fixed-effect model was employed for combined analysis. The results of the meta-analysis demonstrated that patients with recurrent liver cancer who received RH had significantly higher OS compared to those in the TAT group (HR=0.85, 95%CI 0.76∼0.95, P=0.004) (**Figure 2**). Results showed that patients with recurrent liver cancer who underwent RH had significantly higher OS than those in the TAT group.

**Figure 3:**
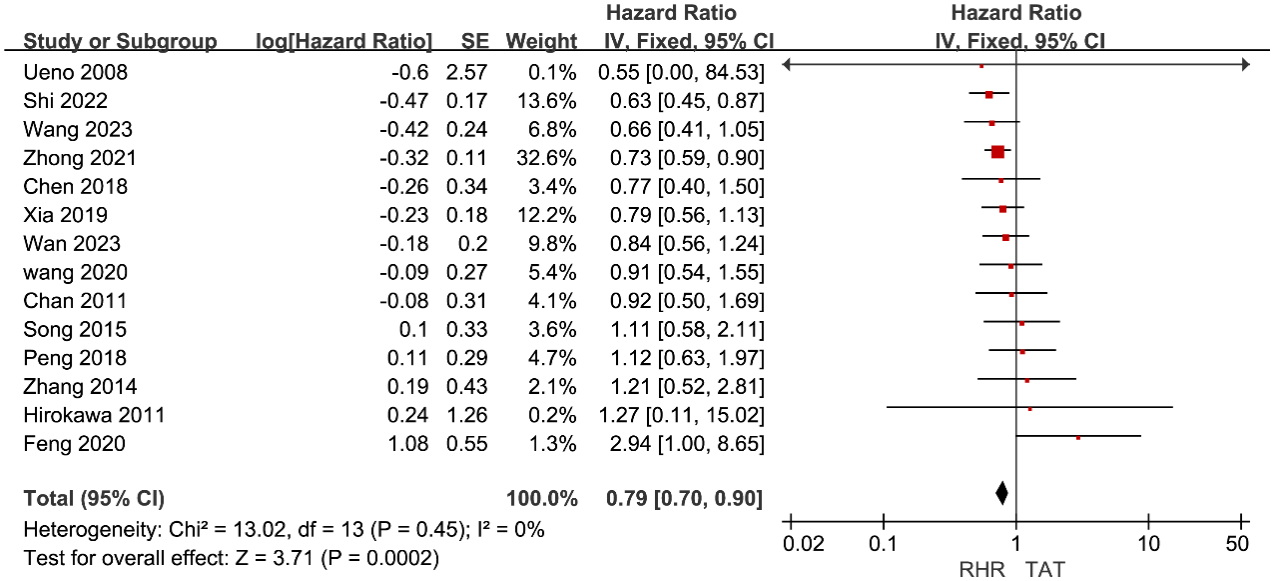
Forest plots of pooled data on recurrence free survival.

The HR data from 14 studies (10–12, 16–18, 27, 28, 31–33, 35, 37, 38) on RFS were included for meta-analysis. Given the low heterogeneity among the study groups (I^2 ^= 0%), a fixed effect model was employed for data integration. The meta-analysis results showed that patients with recurrent liver cancer who underwent RH had significantly higher RFS than those in the TAT group (HR=0.79, 95%CI 0.7∼0.9, P<0.01) (**Figure 3**).

### Postoperative complications and mortality

**Figure 4:**
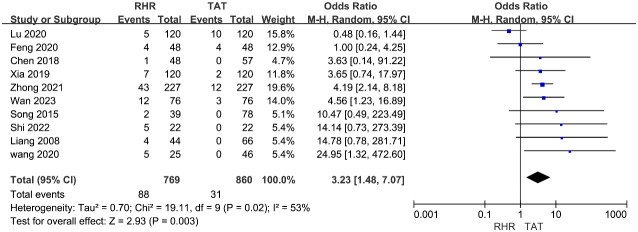
Forest plots of pooled data on severe postoperative complications.

Ten studies (12, 16, 18, 25, 31, 32, 35–38) provided data on severe postoperative complications (Clavien-Dindo grade III or higher).The incidence of severe postoperative complications was 11.4% (88/769) in the RH group and 3.6% (31/860) in the TAT group. The heterogeneity of these trials was moderate (I^2^ = 53%); therefore, a random effects model was employed to pool data. The meta-analysis results revealed a significantly lower incidence of severe postoperative complications in the TAT group compared to the RH group (OR=3.23, 95%CI 1.48∼7.07, P=0.003) (**Figure 4**).

**Figure 5:**
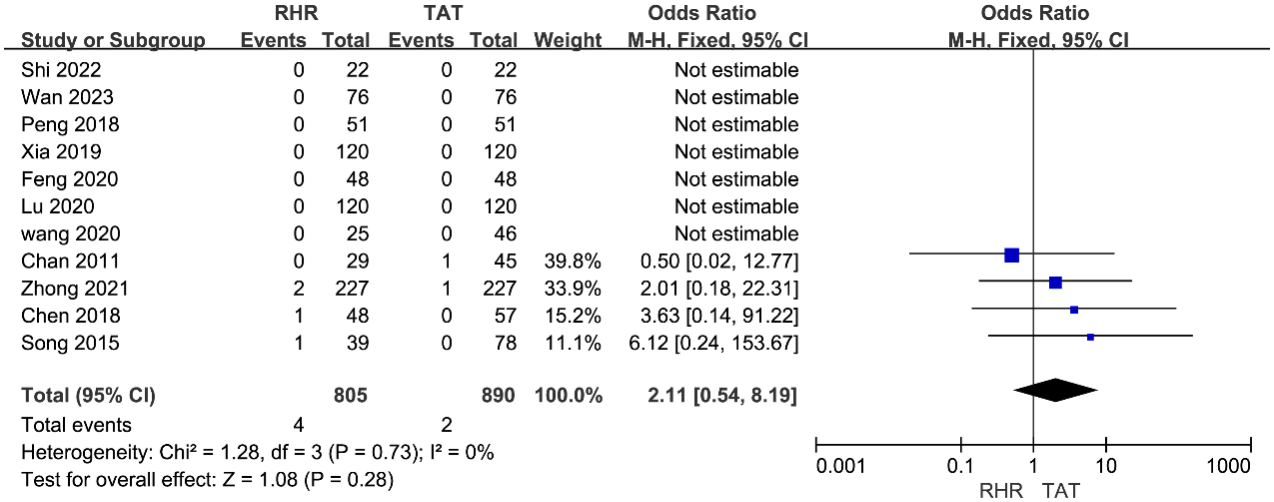
Forest plots of pooled data on mortality.

Eleven studies (12, 16, 18, 28, 31–33, 35–38) provided perioperative mortality. The perioperative mortality of the RH group was 0.5% (4/805), while that of the TAT group was 0.2% (2/890). The heterogeneity of these trials was low (I^2 ^= 48%); therefore, the fixed effect model was used to pool data. Meta-analysis results indicated no statistically significant difference in perioperative mortality between the two groups (OR=2.11, 95%CI 0.54∼8.19, P=0.28) (**Figure 5**).

### Sensitivity analysis and publication bias

**Figure 6:**
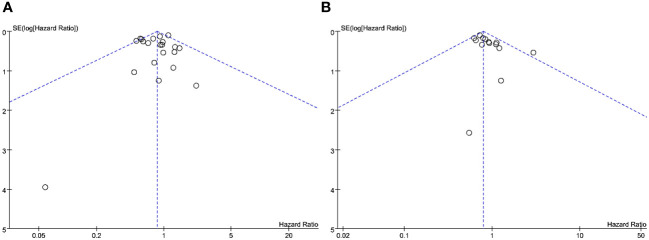
Funnel plot analysis: **(A)** funnel plot of overall survival; **(B)** funnel plot of recurrence-free survival.

Sensitivity analysis was conducted by sequentially excluding individual studies and subsequently performing a pool analysis again. The findings demonstrated that the results of overall survival and recurrence-free survival were basically consistent with the original results, indicating that the meta-analysis results were robust. **Figure 6** illustrates the funnel plot of the included studies. Notably, all plots within the funnel plot display a symmetrical distribution, indicating an absence of discernible publication bias in this meta-analysis.

## Discussion

Recurrence following hepatectomy poses a formidable challenge in the management of HCC (6). When addressing recurrent HCC, it is imperative to concurrently pursue the comprehensive elimination of the tumor and the optimal preservation of residual liver function (5). RHR and TAT stand out as commonly employed modalities for treating recurrent HCC (6). During the initial operation, only a portion of the liver tissue is retained post-hepatectomy, resulting in a significantly diminished liver function reserve compared to the first intervention (12). Additionally, postoperative adhesions pose substantial challenges to reoperation. RHR, with its associated heightened risks of bleeding, infection, and liver failure, exacerbates the complexity of treating recurrent HCC (38). Consequently, some scholars advocate for the utilization of TAT in the management of recurrent HCC, asserting its comparable efficacy to RHR [27]. Literature has reported that only approximately 30% of patients experiencing recurrence after HCC resection have the opportunity for subsequent re-resection, with thermal ablation offering a relatively broad range of applicability (39). Nonetheless, an ongoing debate persists regarding the survival benefits of both RHR and TAT in patients with recurrent HCC (12, 30).

Previous meta-analysis have reported that there was no significant difference between RH and TAT in terms of OS and RFS for patients with recurrent HCC (13). Additionally, RH was associated with higher postoperative complications and mortality (15). However, we conducted a meta-analysis by incorporating newly published studies (16–18) that met the inclusion criteria and re-including data after propensity score matched (16–18, 31, 33, 35–38), which yielded different results from the previous studies. Our findings demonstrate that RH is superior to TAT in terms of OS and RFS in patients with recurrent HCC. This superiority may be attributed to the ability of RH to more thoroughly remove tumor tissue, thereby reducing the risk of residual cancer cells and their spread (18). Moreover, RH proves more effective in controlling local disease, which is crucial for prolonging patient survival time (16). Our sensitivity analysis confirms the robustness of our meta-analysis results, further enhancing the reliability of these findings.

Similarly, it is imperative to acknowledge that TAT represents a technical modality for tumor ablation utilizing high-temperature physical methods, encompassing radiofrequency and microwave ablation techniques (40). TAT possesses distinctive advantages and can be performed via percutaneous, laparoscopic, or open surgery approaches (41). Percutaneous TAT is widely employed in clinical practice as it obviates the need for traditional open surgery (42), thereby reducing patient’s pain and recovery time while enhancing surgical safety. Currently, TAT exhibits extensive applicability across various types and sizes of liver cancer including primary and secondary liver cancer (43). Due to its minimal invasiveness and low postoperative complications, TAT is also regarded as an appropriate treatment option for HCC (12). The findings of this meta-analysis further validate that the perioperative complication rate associated with TAT for recurrent liver cancer is significantly lower compared to that observed with RH. The lower complication rate means patients recover faster and have a shorter hospital stay, making it a potentially safer option for those who can’t handle major surgery.

However, it is important to note that our study did not find any statistically significant difference in perioperative mortality rates between the two treatment modalities. This shows that although surgery and ablation are technically and operationally different, they are both acceptable in terms of safety. Additionally, it should be acknowledged that while targeting the tumor with TAT, there is a possibility of overlooking certain adjacent satellite lesions (44). Hepatectomy can remove both primary tumor lesions and satellite lesions metastasized through portal vein branches (30). Additionally, factors such as tumor morphology, distribution, and ablation range have a much stronger effect on TAT than RH (45). These factors may be the reason why RH is superior to TAT in OS and RFS with recurrent HCC.

The meta-analysis had several limitations. First, almost all the studies included were retrospective studies and only one RCTs was included for evaluation. Therefore, potential confounding factors will reduce the reliability of the meta-analysis results, even if the included study adopts propensity score matching analysis [33]. Second, most of the studies included in the meta-analysis were completed in the Asian region, and the results may be affected by institutional and regional differences. Third, included studies have different surgical indication for recurrent HCC, and the background of the two groups of patients in the same study is inevitably different. Owing to the limitation of data acquisition, this study did not conduct subgroup analysis on tumor size or number, cirrhosis, and recurrence time of recurrent HCC. It is not further clear which patients with recurrent HCC will benefit more from RH. Above reasons may result in a limitation of the conclusion. Therefore, a large sample size, multicenter randomized controlled trial needs to be completed to determine which treatment is most effective for recurrent HCC.

## Conclusion

In conclusion, RH demonstrates a significantly superior survival benefit compared to TAT in the treatment of recurrent HCC. Therefore, in clinical decision-making, RH should be considered as the preferred choice for eligible patients with recurrent HCC. While, it is also necessary to recognize that TAT is an important alternative for the management of recurrent HCC.

## Author contributions

HZ: Writing – review & editing, Writing – original draft, Visualization, Validation, Supervision, Software, Resources, Project administration, Methodology, Investigation, Formal analysis, Data curation, Conceptualization. RD: Writing – original draft, Visualization, Validation, Supervision, Software, Resources, Project administration, Methodology, Investigation, Formal analysis, Data curation, Conceptualization. TZ: Writing – review & editing, Visualization, Validation, Supervision, Software, Resources, Project administration, Methodology, Investigation, Formal analysis, Data curation, Conceptualization. WW: Writing – original draft, Visualization, Validation, Supervision, Software, Resources, Project administration, Methodology, Investigation, Formal analysis, Data curation, Conceptualization.
