# Supplementary material for: Repeat hepatectomy versus thermal ablation therapy for recurrent hepatocellular carcinoma: a systematic review and meta-analysis
Source: Front Oncol. 2024 Mar 28;14:1370390. doi: 10.3389/fonc.2024.1370390 (PMC11007030; doi:10.3389/fonc.2024.1370390)
Supplement: Supplementary file 1 [file Table_1.docx]

| Table S1 Risk of bias for the included studies, based on the ROBINS-I tool | | | | | | | | | |
| --- | --- | --- | --- | --- | --- | --- | --- | --- | --- |
| Author | Year | Type of bias | | | | | | | Overall rating |
|  |  | Confounding | Selection of participants | Exposure assessment | Misclassification during follow-up | Missing data | Measurement of the outcome | Selective reporting of the results |  |
| Choi | 2007 | Low | Moderate | Serious | Moderate | Low | Low | Low | Serious |
| Ueno | 2009 | Low | Low | Moderate | Low | Low | Moderate | Low | Moderate |
| Liang | 2008 | Low | Low | Serious | Moderate | Low | Moderate | Moderate | Serious |
| Umeda | 2010 | Low | Moderate | Low | Low | No information | Low | Low | Moderate |
| Chan | 2012 | Low | Moderate | Low | Low | Low | Low | Low | Moderate |
| Hirokawa | 2011 | Moderate | Low | Moderate | Low | No information | Moderate | Moderate | Moderate |
| Cheng | 2012 | Low | Moderate | Low | Low | Low | Low | Low | Low |
| Zhang | 2014 | Low | Low | Low | Low | Low | Low | Low | Low |
| Wang | 2015 | Low | Low | Serious | Low | Moderate | Low | Low | Serious |
| Song | 2015 | Low | Low | Low | Low | Low | Low | Low | Low |
| Chen | 2018 | Low | Low | Low | Moderate | Low | Low | Low | Moderate |
| Peng | 2018 | Low | Moderate | Low | Low | Low | Low | Moderate | Moderate |
| Xia | 2019 | Low | Low | Low | Low | Low | Low | Low | Low |
| Xiao | 2019 | Low | Low | Low | Low | Low | Low | Low | Low |
| Feng | 2020 | Low | Moderate | Moderate | Moderate | Low | Low | Low | Moderate |
| Lu | 2020 | Moderate | Low | Low | Low | No information | Moderate | Moderate | Moderate |
| Wang | 2020 | Low | Moderate | Low | Low | Low | Low | Low | Low |
| Zhong | 2021 | Moderate | Low | Serious | Low | Moderate | Low | Low | Serious |
| Shi | 2022 | Low | Low | Low | Low | Low | Low | Low | Low |
| Wang | 2023 | Low | Moderate | Low | Moderate | Low | Low | Low | Moderate |
| Wan | 2023 | Low | Moderate | Low | Low | Low | Low | Moderate | Moderate |

ROBINS-I: risk of bias in non-randomized studies of interventions; NA=not applicable
